# Predicting suicidality in late‐life depression by 3D convolutional neural network and cross‐sample entropy analysis of resting‐state fMRI

**DOI:** 10.1002/brb3.3348

**Published:** 2024-01-15

**Authors:** Chemin Lin, Chih‐Mao Huang, Wei Chang, You‐Xun Chang, Ho‐Ling Liu, Shu‐Hang Ng, Huang‐Li Lin, Tatia Mei‐Chun Lee, Shwu‐Hua Lee, Shun‐Chi Wu

**Affiliations:** ^1^ Department of Psychiatry Keelung Chang Gung Memorial Hospital Keelung Taiwan; ^2^ College of Medicine Chang Gung University Taoyuan Taiwan; ^3^ Community Medicine Research Center Chang Gung Memorial Hospital Keelung Taiwan; ^4^ Department of Biological Science and Technology National Yang Ming Chiao Tung University Hsinchu Taiwan; ^5^ Department of Engineering and System Science National Tsing Hua University Hsinchu Taiwan; ^6^ Department of Imaging Physics University of Texas MD Anderson Cancer Center Houston Texas USA; ^7^ Department of Head and Neck Oncology Group Linkou Chang Gung Memorial Hospital and Chang Gung University Taoyuan Taiwan; ^8^ Department of Diagnostic Radiology Linkou Chang Gung Memorial Hospital and Chang Gung University Taoyuan Taiwan; ^9^ Department of Psychiatry Linkou Chang Gung Memorial Hospital Taoyuan Taiwan; ^10^ Laboratory of Neuropsychology and Human Neuroscience The University of Hong Kong Pok Fu Lam Hong Kong; ^11^ State Key Laboratory of Brain and Cognitive Science The University of Hong Kong Pok Fu Lam Hong Kong

**Keywords:** convolutional neural network, cross‐sample entropy, machine learning, older adult, resting‐state fMRI, suicide, suicide attempt

## Abstract

**Background**: Predicting suicide is a pressing issue among older adults; however, predicting its risk is difficult. Capitalizing on the recent development of machine learning, considerable progress has been made in predicting complex behavior such as suicide. As depression remained the strongest risk for suicide, we aimed to apply deep learning algorithms to identify suicidality in a group with late‐life depression (LLD).

**Methods**: We enrolled 83 patients with LLD, 35 of which were non‐suicidal and 48 were suicidal, including 26 with only suicidal ideation and 22 with past suicide attempts, for resting‐state functional magnetic resonance imaging (MRI). Cross‐sample entropy (CSE) analysis was conducted to examine the complexity of MRI signals among brain regions. Three‐dimensional (3D) convolutional neural networks (CNNs) were used, and the classification accuracy in each brain region was averaged to predict suicidality after sixfold cross‐validation.

**Results**: We found brain regions with a mean accuracy above 75% to predict suicidality located mostly in default mode, fronto‐parietal, and cingulo‐opercular resting‐state networks. The models with right amygdala and left caudate provided the most reliable accuracy in all cross‐validation folds, indicating their neurobiological importance in late‐life suicide.

**Conclusion**: Combining CSE analysis and the 3D CNN, several brain regions were found to be associated with suicidality.

## Graphical abstract

Predicting suicide in older adults is difficulty. Using machine learning, we can predict suicidality from the certain brain regions' complexity of the resting state fMRI data.

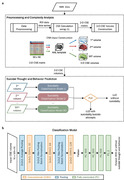

## 1 INTRODUCTION

With population aging, two billion older adults were estimated by 2050 (Harper, 2014), making late‐life suicide a growing concern in public health (De Leo, 2022). Study has shown that depression remained the strongest risk factor for late‐life suicide, along with poor perceived health, poor sleep quality, and limited close relatives or friends (Turvey et al., 2002). However, predicting suicide has always been difficult. A recent meta‐analysis has suggested that the prediction accuracy of the traditional risk factor analysis for the last 50 years is only marginally superior than chance and that machine learning‐based algorithm could outperform the traditional way, as it can deal with complex interplay among multiple predictors, a scenario often found in suicide (Franklin et al., 2017). Apart from clinical predictors, people have resorted to plausible biological markers, but existing biological risk factors only serve as weak predictors for future suicidal behavior (Chang et al., 2016). Novel neuroimaging has emerged as a potential marker for its capability to identify brain alterations associated with suicidal behavior in vivo and to develop targeted preventive strategies.

Studies on neuroimaging have found that the brain regions responsible for emotion and impulse control, including the ventral or dorsal prefrontal cortex, insula, mesial temporal lobes, striatum, and posterior structures, are associated with suicide‐related thoughts and behavior across different mental disorders (Schmaal et al., 2020). The orbitofrontal cortex and dorsolateral prefrontal cortex (DLPFC) were particularly associated with suicide for their involvement in decision‐making, problem solving, and fluency (Van Heeringen et al., 2011). In patients with late‐life depression (LLD) with past suicide attempts, a study on functional magnetic resonance imaging (fMRI) demonstrated a weak ventromedial PFC response to expected reward in a probabilistic reversal learning task (Dombrovski et al., 2013). In addition, suicide attempters with LLD had smaller putamen gray matter volume, indicating the need for immediate reward in a gambling task (Dombrovski et al., 2012). These prior studies have demonstrated that neuroimaging can decipher specific brain regions related to the emotional and cognitive feature of suicidal behavior.

Similar to research on suicide, studies on neuroimaging have also embraced machine learning in recent years, as machine learning can exploit the richness of the data in both fields. Imaging analysis benefits from machine learning by using an algorithm to recognize patterns and features in magnetic resonance imaging (MRI) data (Mateos‐Pérez et al., 2018). Recently, traditional machine learning has evolved to deep learning, which uses multiple hidden neural networks to learn complex and abstract features in a hierarchical way. Using nonlinear transformations, deep learning can discover more complex and abstract patterns in data (Vieira et al., 2017), which is important to suicide studies where brain changes are often subtle to detect (Schmaal et al., 2020). At present, a convolutional neural network (CNN) is a state‐of‐the‐art tool among deep learning methods because of its ability to capture delicate feature representation during imaging (Segato et al., 2020). Using a CNN, diagnosing Alzheimer's disease (Duc et al., 2020), attention‐deficit hyperactivity disorder (Ariyarathne et al., 2020), or schizophrenia (Qureshi et al., 2019) by only using resting‐state fMRI data has become attainable. Moreover, using a three‐dimensional (3D) CNN, the algorithm can fully exploit the spatial information in the resting‐state fMRI data to achieve disease classification (autism vs. healthy subjects) and age prediction (Khosla et al., 2019). Table 1 presents a comprehensive summary, including the diseases under consideration, the utilized machine learning and deep learning methodologies, the modalities of input data, and the respective accuracies from these studies.

**TABLE 1:** A summary of the accuracy of the referenced studies using machine learning and deep learning methods.

Diseases	Methods	Input modalities	Accuracy
Alzheimer's disease Duc et al. (2020), Mateos‐Pérez et al. (2018), and Vieira et al. (2017)	SVM, OPLS, random forests	T1, PET, DTI, CSF	0.82–0.965
	CNN, AE, and CNN	rs‐fMRI	0.80–0.969
	3D CNN	rs‐fMRI	0.8527
Autism Mateos‐Pérez et al. (2018)	SVM, decision tree, LDA, QDA	T1, DTI, spectroscopy	0.70–0.963
Multiple sclerosis Mateos‐Pérez et al. (2018)	SVM, logistic regression	T1, T2	0.871–0.96
Parkinson's disease Mateos‐Pérez et al. (2018)	SVM, bootstrap, multinomial logit	T1, T2, DTI	0.42–0.927
Attention deficit hyperactivity disorder Ariyarathne et al. (2020), Mateos‐Pérez et al. (2018), and Vieira et al. (2017)	SVM, Gaussian process classifier	T1	0.793 – 0.902
	AE and CNN, FCC,^a^ DBN,^a^ DBaN^a^	rs‐fMRI	0.344–0.95
	CNN based on extracted seed correlations	rs‐fMRI	0.84–0.86
Depression Mateos‐Pérez et al. (2018)	SVM	T1, DTI	0.70–0.831
Mild cognitive impairment Vieira et al. (2017)	DAE,^a^ SAE	rs‐fMRI	72.6–87.5
Schizophrenia Qureshi et al. (2019), Vieira et al. (2017)	SAE	rs‐fMRI	85.8
	3D CNN	rs‐fMRI	0.98 ± 0.01
Early detection of brain tumor Segato et al. (2020)	MRI	CNN, SVM	0.98
Volumetric assessment of meningiomas Segato et al. (2020)	MRI‐T1WI MRI‐T2WI	CNN, FCNN	0.81 (DSI)
Segmentation of brain tumor Segato et al. (2020)	MRI‐T1WI MRI‐T2WI	CNN, FCNN	0.86 (DSI)

*Note*: Accuracy represents the ratio of true outcomes, including both true positives and true negatives within *a* test.

Abbreviations: AE, autoencoder; CNN, convolutional neural network; DAE, deep autoencoder; DBN, deep belief network; DBaN, deep Bayesian network; DSI, dice similarity index; FCC, fully connected cascade; OPLS: orthogonal partial least squares; QDA: quadratic discriminant analysis; SAE: stacked autoencoder.

^a^ Incorporating feature selection.

Thus, we aimed to predict suicidality in older adults with depression by using 3D CNN on resting‐state fMRI data. Based on our previous studies, we will explore the nonlinear property in fMRI data in each brain region by examining its cross‐sample entropy (CSE) value, a measurement of signal complexity among brain regions (Chen et al., 2020; Lin et al., 2019). We had successfully used 3D, CNN, and CSE analysis on resting‐state fMRI data to classify patients of LLD from controls (Lin et al., 2023). We also sought to determine the brain regions with the highest accuracy to differentiate patients of LLD with suicide‐related thought and behavior from counterparts with non‐suicidality. We expect that our findings could extend our knowledge of the neural basis in late‐life suicidality.

## 2 MATERIALS AND METHODS

### 2.1 Participants

We recruited participants who were more than 60 years old and had at least one DSM‐5 (American Psychiatric Association, 2013) diagnosis of major depressive disorder after age 55 (i.e., LLD), regardless of the onset age of their first depressive episode. A diagnostic interview was conducted by two board‐certified geriatric psychiatrists (Lin and Lee). Patients were recruited from psychiatric service in a tertiary medical center. Disease course and suicide history were probed by using Mini‐international neuropsychiatric interview (Lecrubier et al., 1997). Based on current suicide ideation and past history of suicide attempt, patients were divided into patients with suicidality and LLD group without suicidality. Patients with suicidality included those with suicide ideation and suicide attempter (i.e., inclusive of those who had serious desire to die and those who had ever attempted suicide). Suicide attempt was defined as past self‐harm behavior with the intention to die. The LLD group without suicidality referred to those who had no suicide ideation and no past history of suicide attempt.

Depression severity and suicide intent were evaluated using the 17‐item Hamilton Depression Rating Scale (Hamilton, 1960) and Beck Scale for Suicide Ideation (Beck et al., 1979), respectively. During the study period, all the patients kept their psychotropics with the same dosage maintained for at least 2 months because of ethical reasons. The Antidepressant Treatment History Form was administered to gage the refractoriness of patients to treatment (Sackeim et al., 2019). Except for the comorbidity of anxiety disorder, patients were excluded if they met other DSM‐axis I major psychiatric diagnosis or had history of head trauma, stroke, major neurocognitive decline, Parkinson's disease, thyroid dysfunction, and other major neurological disorders. Patients all had a minimum score of 24 in Mini‐Mental State Examination (Folstein et al., 1975). We focus solely in LLD as depression is the major risk factor in late‐life suicide. Moreover, we try to avoid confounding factors of other types of mental illness. All participants signed an informed consent that indicates the study protocols approved by the institutional review board of the Chang Gung Medical Foundation (IRB No. 201202970B0C601).

### 2.2 Data acquisition

We collected our MRI data using an eight‐channel head coil on a 3T MRI scanner (Discovery MR750, GE Healthcare). Participants were asked to keep their eyes closed, not to think of anything, and not to fall asleep during the scan. Resting‐state functional MRI data were collected using a *T*2*‐weighted gradient‐echo echo‐planar imaging sequence using the following parameters: repetition time (*T*R) = 2000 ms, echo time (TE) = 30 ms, flip angle = 90°, number of slices = 36, in‐plane matrix size = 64 × 64, and slice thickness = 4 mm. A total of 180 dynamic volumes were acquired for each subject. *T*1‐weighted structural images were acquired using an with IR‐prepared 3D spoiled gradient echo sequence with: TR = 8.184 ms, TE = 3.2 ms, inversion time (TI) = 450 ms, flip angle = 12°, FOV = 250 × 250 mm^2^, voxel size = 0.98 × 0.98 × 1 mm^3^, and slice number = 160.

### 2.3 Image preprocessing

Preprocessing included the following steps: slice‐timing correction, motion correction by realigning images to the first volume and removing images showing 2 mm axial displacement or 2° rotation angle, normalization and deformation to Montreal Neurological Institute template, and reslicing to 2 × 2 × 2 mm^3^ isotropic voxel dimensions. These procedures were implemented using SPM12 (statistical parametric mapping, http://www.fil.ion.ucl.ac.uk/spm/ ). Further preprocessing steps used the REST toolbox (http://restfmri.net/forum/REST_V1.8 ). At each voxel, the time series were detrended and bandpass filtered (frequencies between 0.01 and 0.08 Hz). The time courses for various covariates (white matter, cerebrospinal fluid, and six motion parameters for head movement) were extracted and regressed out as nuisance regressors to eliminate potential effects of physiological artifacts. Finally, the gray matter of the brain was divided into 90 regions of interests (ROIs) based on automated anatomical labeling (AAL) (Tzourio‐Mazoyer et al., 2002). The data time series of all voxels in an ROI were averaged. The brain networks were visualized using the BrainNet Viewer toolbox (http://www.nitrc.org/projects/bnv/) (Xia et al., 2013).

### 2.4 Proposed scheme

**FIGURE 1:**
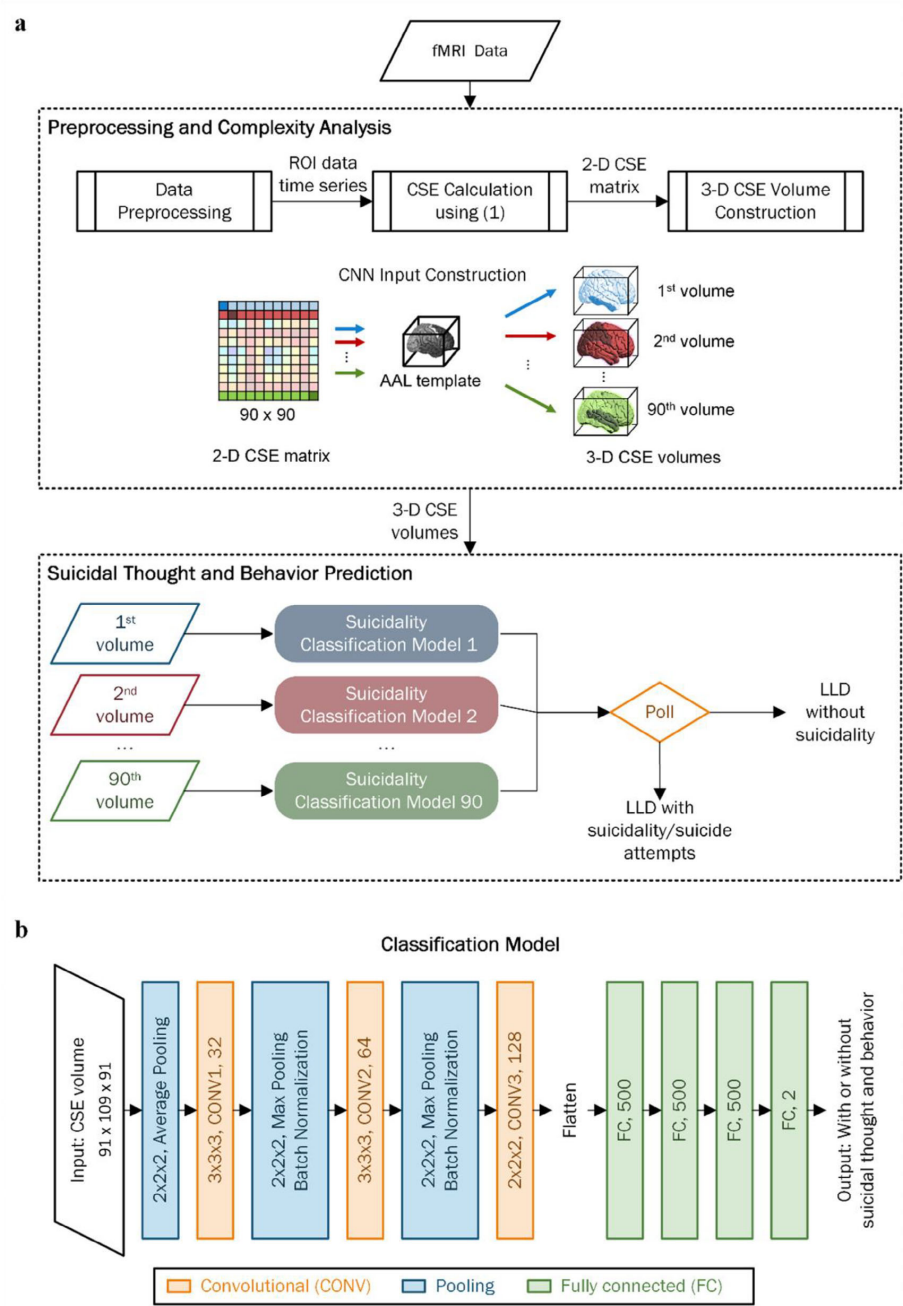
(a) Flow diagram of the proposed scheme for suicidal thought and behavior prediction. (b) The architecture of the proposed network model for subject classification.

The flow of the proposed scheme for suicidal thought and behavior prediction is shown in Figure 1a, with the 3D CSE volume and CNN for subject classification being the two major components. The CSE matrix shown in Figure 1a is a 90 × 90 matrix containing the CSE value of all AAL ROI pairs. The following equation was used to calculate the CSE between any ROI pair (Gómez et al., 2009; Richman & Moorman, 2000):

(1) $$\mathrm{CSE}\;\left(m,r,L\right)=\;-\ln\frac{p^{m+1}}{p^{m}},$$

where $p^{l}=\;1/\left(L-1\right)\sum_{i\;=\;1}^{L-1}p_{i}^{l}$ with l (*m* or *m* + 1 in Equation (1) with *m* = 2 in this study) being the length of two sub‐vectors $x_{l}\left(i\right)\;=\;\left[x_{i},\;x_{i+1},\text{…},x_{i+l-1}\right]$ and $y_{l}\left(j\right)\;=\;\left[y_{j},\;y_{j+1},\text{…},y_{j+l-1}\right]$ from the data series **x** and **y** of an ROI pair. The parameters $p_{i}^{l}=n_{i}^{l}\;/\left(L-1\right)$ and $n_{i}^{l}$ represent the probability and number of vectors, in which any *l*‐point sub‐vector $y_{l}\left(j\right)$ in **y** matches the *l*‐point sub‐vector $x_{l}\left(i\right)$ in **x**. The indices *i* and *j* vary from 1 to L−1. A match with a tolerance r is defined as $d[x_{l}\left(i\right),y_{l}\left(j\right)]<r$ with

(2) $$d\;\left[x_{l}\left(i\right),y_{l}\left(j\right)\right]=\;\max\left\{\left|x_{i+k}-j_{j+k}\right|:0\leq k\leq l-1\right\},$$

which is the maximum difference between the components of $x_{l}\left(i\right)$ and $y_{l}\left(j\right)$. In this study, the tolerance parameter *r* was set as.6. The *i*th row of the CSE matrix contains the CSE value of the 90 ROIs with regard to the *i*th ROI. By assigning the entries of CSE matrix's *i*th row to the voxels of their corresponding ROIs (Chen et al., 2020), we can construct a 91 × 109 ×91 3D CSE volume seeded at the *i*th ROI. Each entry in a CSE matrix was centered and scaled on the basis of the mean and standard deviation of the CSE matrices of the subjects without suicidal thought and behavior before CSE volume construction. Finally, each subject has 90 CSE volumes to feed into different classification network models. A CSE volume provides detailed brain interactions from temporal and spatial aspects.

The architecture of the network model for suicidal thought and behavior classification is shown in Figure 1b. For any subject under test, the network takes one of their 3D CSE volumes to determine whether or not they have suicidal thoughts and behavior. The network starts with an average‐pooling layer to reduce data dimensionality. Two of the three convolution layers are followed, each with intervening max‐pooling and batch normalization layers. Afterward, the third convolutional layer was connected, whose output was fed to four successive fully connected layers. The number of neurons is 500 in the first three fully connected layers and two in the last layer. The convolution layers had 32, 64, and 128 filters of sizes 3 × 3 × 3, 3 × 3 × 3, and 2 × 2 × 2, respectively. The filter sizes for average pooling and two max‐pooling layers were all 2 × 2 × 2. All trainable layers were followed by the ReLU activation function, except for the last fully connected layer, where the softmax function was used for classification purposes. One difficulty associated with the seed‐based method for brain functional connectivity analysis is that no standard has been established for selecting seed ROIs, and the selection is often different across studies (Cole et al., 2010; Sohn et al., 2015). We polled the classification results from all the 90 classification models trained on the CSE volume seeded at 90 ROIs to comprehensively investigate the effect of different ROIs on the prediction of suicidal thought and behavior and refrained from the need of prior knowledge for ROI selection. This ensemble learning strategy (Sagi & Rokach, 2018) that integrates different ROI selections could predict suicidal thought and behavior.

### 2.5 Model training

We enrolled 83 participants, including 48 and 35 patients of LLD with suicidal ideation or past suicide attempts, respectively. We employed *k*‐fold cross‐validation to assess the model's ability to perform well on unseen data, measuring its generalization error. This widely adopted technique provides a more comprehensive evaluation by utilizing diverse data subsets for training and testing, in contrast to a single train‐test split (Mohri et al., 2018). We randomly divided the participants into six roughly same‐size groups to perform cross‐validation (Mohri et al., 2018). Although the data partition was conducted in a “random” manner, specific criteria were introduced during this process to ensure that individuals with histories of prior suicide events are included in all folds. To elaborate, we have taken steps to evenly distribute three distinct groups of individuals—namely, patients of LLD without suicidality, with suicide ideation, and with suicide attempt—across all folds, thereby ensuring their representation in each fold. One participant group was retained as the testing dataset during each CV process to provide an unbiased evaluation of a final model evaluation on the training and validation datasets. Although only onefold was designated as the test set for performance evaluation during each iteration, the assessment of its generalization ability encompassed contributions from all participants, not limited solely to the 13 or 14 participants. Among the five remaining participant groups, four were used to train the network model, and one was used to estimate the generalization error of the model acquired from the training set to avoid model overfitting. These three datasets are normally seen in deep learning applications. We used the categorical cross‐entropy loss for classification during model training, which was optimized using the adaptive moment estimation optimizer with an epoch size of 400. We adopted a batch size of 32, aligning with the previous recommendations (Bengio, 2012; Kandel & Castelli, 2020). Given that the datasets are biological images (e.g., fMRI), a batch size of 32 has established itself as the preferred default choice. Considering our utilization of a small batch size, we opted for a modest learning rate of 10^−3^ based on previous recommendation (Kandel & Castelli, 2020). If the loss did not show improvement within 60 epochs, the training procedure was stopped. The CV process was repeated six times, with each of the six participant groups used exactly once as the testing dataset. All the models were trained in TensorFlow 1.2.1 using the CUDA 11.1 Toolkit and cuDNN v8.0.5 on the computing platform: ASUS ESC8000 G4 server system with Intel Xeon CPU, GeForce RTX 2080 Ti, and 192 GB RAM.

## 3 RESULTS

Table 2 presents the demographic and clinical data of the participants. A total of 83 patients with LLD were recruited, 35 of which were non‐suicidal and 48 were suicidal. Among the 48 patients with suicidality, 26 had only suicidal ideation and 22 had past suicide attempts. Compared with patients without suicidality, patients with suicidality had an early depression onset with more lifetime depressive episodes, a difference mostly driven by those with suicide attempt (Table S1).

**TABLE 2:** Demographic data and between group comparisons.

	NS	Suicidality	Statistics
	(*n* = 35)	(*n* = 48)	
Age	67.2 ± 5.8	65 ± 4.8	*t* = 2.0
Sex, (M/F)	7/28	8/40	Chi = 0.25
Education	7.9 ± 2.9	9.2 ± 3.6	*t* = −1.8
Disease course			
Onset	57.1 ± 7.9	50.4 ± 11.6	*t* = 2.9*
Episodes	1.9 ± 1.6	3.3 ± 2.9	*t* = −2.4*
ATHF load	3.4 ± 1.2	3.8 ± 1.1	*t* = −1.5
Psychological scales			
HAMD	7.6 ± 4.7	9.4 ± 6.4	*F* = 2.5
BSS	2.4 ± 2.7	6.3 ± 5.6	*F* = 9.4**
MMSE	27.7 ± 1.5	27.9 ± 1.3	*F* = 0.5

Abbreviations: ATHF, antidepressant treatment history form; BSS, Beck Scale for Suicide Ideation; HAMD, 17‐item Hamilton Depression Scale; MMSE, Mini‐mental Status Examination; NS, non‐suicidal late‐life depression.

*p < .05.

**p < .005.

**FIGURE 2:**
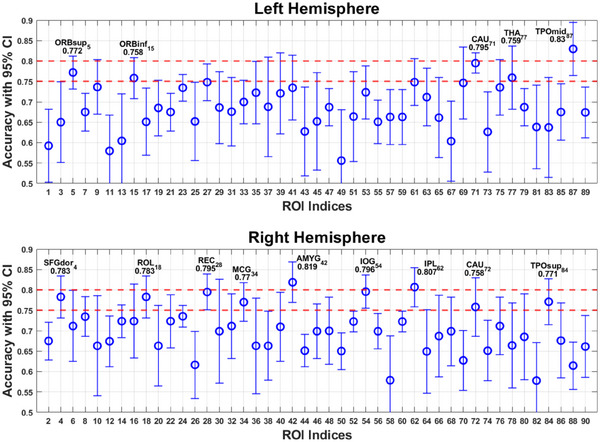
The mean classification rates with 95% confidence interval of different regions of interests (ROIs) in left and right hemispheres. AMYG, amygdala; CAU, caudate; IOG, inferior occipital gyrus; IPL, inferior parietal lobule; MCG, middle cingulate gyrus; ORBsup, inferior orbitofrontal cortex; ORBinf, inferior orbitofrontal cortex; ROL, rolandic operculum; REC, rectus gyrus; SFGdor, dorsal superior frontal gyrus; THA, thalamus; TPOmid, middle temporal pole; TPOsup, superior temporal pole.

Five of the six testing datasets had 14 participants, and the remaining one had 13. The classification rate attained by different ROI classifiers varied during testing, with their classification rates averaged over six CV processes (Figure 2). We also computed the 95% confidence intervals (Bayle et al., 2020) in each ROI. It is worthy noting that all the ROIs of accuracy rate above 75% had narrow lower bound of their confidence intervals that exceed 70%. This not only reinforces the links between these ROIs and suicidality but also underscores the consistency observed across different data folds.

We detailed the results of the six cross‐validations in Table S2 where 11 patients without suicidality were mis‐classified as having suicidality in the model.

**FIGURE 3:**
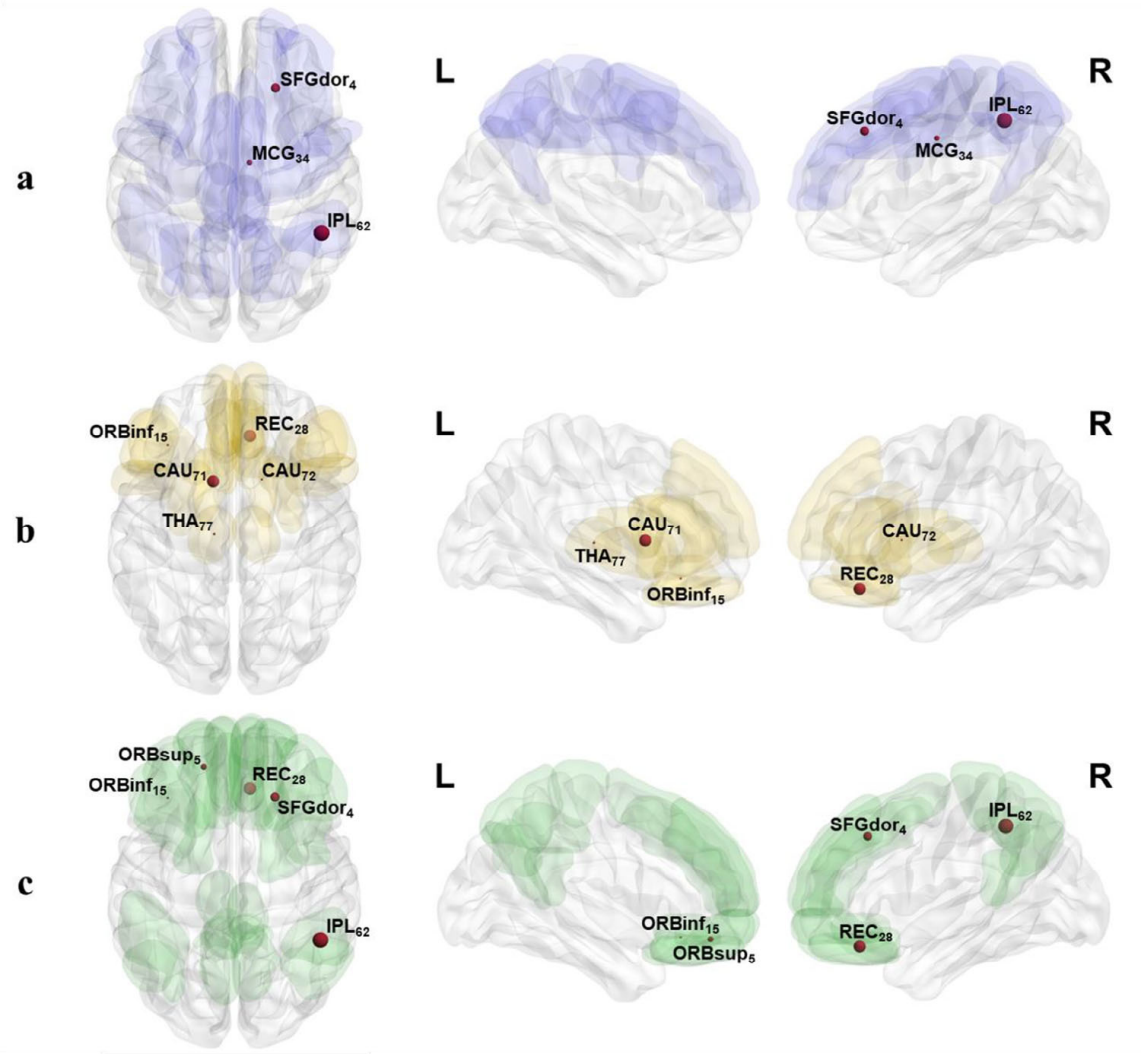
Regions of interests (ROIs) whose classifiers achieved a more than 75% classification rate: (a) in the fronto‐parietal network; (b) in the cingulo‐opercular network; and (c) in the default mode network.

After 6 rounds of cross‐validation, we obtained a mean accuracy rate above 75% in the classifiers of 14 ROIs. These ROIs were located in default‐mode network (DMN; orbital part of the left superior frontal gyrus and right rectus gyrus), fronto‐parietal network (FPN; the dorsolateral part of the right superior frontal gyrus, right midcingulate gyrus, and right inferior parietal lobule [IPL]), cingulo‐opercular network (CON; the orbital part of the left inferior frontal gyrus, left thalamus, and caudate), and in regions outside the three major resting‐state networks (right rolandic operculum, right amygdala, right inferior occipital gyrus, temporal poles in the right superior temporal gyrus, and left middle temporal gyrus; Figure 3). Besides reaching a mean accuracy above 75%, the right amygdala and left caudate also obtained this accuracy in every one of the six cross‐validation processes, indicating that they were the most reliable nodes to classify suicidality.

## 4 DISCUSSION

In the present study, we could separate patients of LLD with suicidality from those without by 3D convolution neural networks. The CSE of the brain regions that are most predictive of suicidal behavior resided primarily in three canonical resting‐state networks, including DMN, FPN, and CON, with a mean accuracy rate above 75%. Moreover, the machine learning models using fMRI data from the right amygdala and left caudate provided the most reliable accuracy across all six cross‐validation processes, indicating their unique neuropathological roles in late‐life suicide.

Our finding is consistent with previous meta‐analysis showing that the right amygdala is consistently associated with suicidal thoughts or behavior (Huang et al., 2020). The right amygdala subserves implicit and autonomic emotional regulation compared with the left amygdala (Gläscher & Adolphs, 2003; Williams et al., 2005). Emotional dysregulation, partly resulting from early‐life adversity, is a major trait in suicide (Turecki, 2014). Although prior studies on emotion dysregulation have focused on adolescents and adults (Colmenero‐Navarrete et al., 2021), our studies suggest that older adults may share the same psychological theory. Caudate is another brain region in our study showing high predictive accuracy in suicide prediction. Increased prodynorphin mRNA expression level, an opioid gene expression, was found in the caudate of patients with suicidality (Hurd et al., 1997). The opioid system is implicated in suicidal behavior, as physical pain and social pain are tightly related (Lutz et al., 2017). However, the putamen showed volume reduction in late‐life suicide compared with the caudate (Dombrovski et al., 2012). By contrast, another study showed that increased caudate volume was associated with violent suicidal behavior (Jollant et al., 2018). Nevertheless, these inconsistent results were all derived from structural MRI studies. Another recent functional fMRI study has found that the functional connectivity and directionality from the caudate to the ventrolateral prefrontal cortex is a characterizing feature in suicidal ideation and behavior in LLD (Shao et al., 2021). In addition, analyzing resting‐state fMRI data using a linear (Shao et al., 2021) or nonlinear (such as the entropy analysis in the current study) approach, the caudate was found to be a critical brain area associated with suicidal behavior in LLD. Caudate is the center of the cortico‐basal ganglia‐thalamocortical system. Our current study also found the inferior frontal gyrus (orbital part) and thalamus to be highly predictive of suicidality, which are two regions in this circuit. The cortico‐basal ganglia‐thalamocortical system is responsible for inhibitory control, decision‐making, and working memory (Wei & Wang, 2016), whose malfunction may lead to suicidality (Jollant et al., 2011; Richard‐Devantoy et al., 2012). Moreover, the caudate is connected to the dorsolateral prefrontal cortex (DLPFC) (Graff‐Radford et al., 2017), which is the dorsolateral part of the superior frontal gyrus. Thus, the caudate could be a pivotal area implicated in late‐life suicide. It is one of the two regions (along with the amygdala) that consistently show high predictive accuracy in all cross‐validation models.

DLPFC dysfunction has been a consistent finding in neuroimaging studies on suicide, as the consequential top‐down behavioral disinhibition and diminished flexibility may initiate the transition from suicidal ideation to behavior (Schmaal et al., 2020). Our finding of the right, but not the left, DLPFC, is consistent with previous studies on suicide attempters with schizophrenia (Matsuoka et al., 2020) or those with a family history of suicide (Ding et al., 2017; Jollant et al., 2018). Compared with the left DLPFC, the right DLPFC plays a role in actual mental generation while considering the interdependence among sequences in action planning, a phenomenon observed in the study using the Tower of London test, where intermediate moves are required (Kaller et al., 2010). In the lesion study, the right DLPFC is necessary not only to manipulate working memory, but also to broaden reasoning (Barbey et al., 2013). Previously, impairment in executive function, particularly the cognitive control in attaining certain goals, has been found to play a critical role in suicidal ideation in older adults (Gujral et al., 2014). Our finding of the right DLPFC and the function it subserves could infer a specific aspect of the executive dysfunction associated with late‐life suicide. Interestingly, repetitive transcranial magnetic stimulation (rTMS) to the left prefrontal cortex has been shown to have anti‐suicidal effect in patients with mid‐life depression (George et al., 2014). Our finding of right DLPFC could be a potential target of rTMS for patients of late‐life suicide.

The orbital part of the superior frontal gyrus is another region predictive of suicidality in our study. Gosnell et al. (2019) found that the decreased functional connectivity between this region and other regions within the “hate circuitry” can classify patients with suicidal behavior through machine learning. They hypothesized that “hate” may be self‐referential to precipitate suicidal behavior. Similarly, our findings of the orbital part of the superior frontal gyrus, rectus gyrus, and IPL all reside in the DMN, a network performing self‐referential and rumination (Hamilton et al., 2015). Another research has found that decreased functional connectivity between FPN (particularly IPL) and DMN was associated with higher suicide risk (Dai et al., 2022). Therefore, our finding of nodes predictive of suicidality in DMN and FPN can be explained by this inter‐network functional connectivity change, which results in impaired top‐down emotional control.

The right rolandic operculum and bilateral temporal poles are other brain regions predictive of suicidality outside the triple resting networks. Similarly, Gosnell et al. (2019) also found that the resting‐state functional connectivity increase in the rolandic operculum and decrease in the temporal pole were associated with suicidality. Hypoperfusion in the rolandic operculum has been observed in suicide completers from a cohort study (Willeumier et al., 2011). Other study has found that the functional connectivity between the middle temporal pole and rostral anterior cingulate cortex was negatively correlated with the severity of suicidal ideation in patients with depression (Du et al., 2017). The Rolandic operculum and temporal pole are responsible for the integration of perceptual inputs, including interoceptive or visceral emotional signals (Olson et al., 2007; Triarhou, 2021). Suicide attempters exhibited interoceptive numbing; thus, they can override the painful consequence of self‐injury (DeVille et al., 2020). In addition, the temporal pole can integrate information from various modalities, and it is associated with social cognition and emotion processing (Pehrs et al., 2015). In suicide attempters with LLD, impairment in social cognition was found to be associated with difficulty in interpersonal relationship and poor social support, which all undermined suicidal behavior (Szanto et al., 2012). Thus, our finding of the right middle temporal pole having the highest mean classification rate in predicting suicidality suggests that social cognition dysfunction could be an important factor affecting late‐life suicide.

Our results were drawn from entropy analysis on the resting‐state fMRI. This nonlinear temporal measure of the fMRI data can reflect the scale‐free property of the resting brain (Pritchard et al., 2014) and correlate well with neurocognitive function (i.e., brain reserve) (Wang, 2021) and human intelligence (Saxe et al., 2018). In addition, different brain networks can be discerned on the basis of their entropy values during rest or working memory test (Nezafati et al., 2020). Our results further show that the CSE from the three resting‐state networks can be used to predict suicidality in LLD. Using entropy to quantify the temporal variability during neural processing can reveal brain's ability to adapt to the changing environment (Keshmiri, 2020). Failure to adapt to or resolve real‐life difficulties could result in late‐life suicide, as study showed that older adults who attempted suicide often perceived life problems as threatening and unsolvable (Gibbs et al., 2009). Thus, behavior change highly correlates with the temporal variability of brain's activity (Keshmiri, 2020). Our results support this notion by demonstrating entropy measurement, which served as a marker to explore suicidality in older adults.

This study has a few limitations. First, our sample size was small, which would impede us from having a more fine‐grained classification of suicidal behavior in our participants (e.g., planned vs. unplanned suicide) (Bernanke et al., 2017). However, suicide encompasses various phenotypes in the clinical setting. Therefore, traditional theoretically driven models fail to outperform machine learning models to predict suicidality because of the complexity and heterogeneity in suicide (Schafer et al., 2021). However, our results obtained from using a machine learning approach could provide potentially unifying neural substrates of suicidality in LLD. Moreover, one recent study using generalized q‐sampling imaging with CNN‐based model could distinguish 41 depressive patients with suicidal ideation from 58 patients without suicidal thoughts with a prediction accuracy of 85% (Weng et al., 2020). This suggests that our sample size is adequate to build a suicide prediction algorithm. However, it is still suggested to increase the sample size in developing machine learning algorithm from brain MRI data to avoid risk of bias (preferable with sample sizes more than 20 in contrast to the number of candidate features) (Parsaei et al., 2023). Second, we combined the group of suicidal ideation and suicide attempters into one group of suicidality. Suicide attempts and suicidal ideation are considered the strongest predictors for suicide death (Szanto et al., 2002). Among older adults, suicidal ideation is less expressed compared with other age groups (Conwell et al., 1998). They also had the lowest ratio of suicide attempts to completion due to their high intention to end their lives. Therefore, when they express their suicidality, either through suicidal ideation or attempt, clinicians and health providers should be at high alert to halt the progression to suicide completion. Thus, we treated both conditions as one entity in the classification model. Third, although our machine learning results are promising, we did not perform external validation (Dwyer et al., 2018). However, the testing dataset in our model was preserved to provide unbiased model evaluation. Although our study achieved a mean accuracy rate above 75% in classifying patients of LLD without or with suicidality, it is important to note that a significant proportion of errors occurred, primarily involving the misclassification of patients of LLD without suicidality as suicidal. To address this issue, one potential avenue is the exploration of a classification model capable of revealing discriminative information to distinguish between the three distinct patient groups. However, it is essential to acknowledge that training such a model presented challenges due to the limited number of patients available for our study. Moreover, our use of *k*‐fold cross‐validation (internal validation) provides adequate accuracy without inflating the results (Jacobucci et al., 2021). Furthermore, we propose the extrapolation of our models to another dataset or in other culture in the future.

## 5 CONCLUSION

Suicide has been the most elusive and devastating health problem in psychiatry, which is lethal for older adults. Translating the advances of machine learning such as deep learning in neuroimaging data into psychiatric application is still in its infancy (Walter et al., 2019). Although machine learning can identify those who are at risk, how to develop a scalable intervention afterward is even more critical (Kirtley et al., 2022). Moreover, machine learning often yields complex algorithms that are difficult in clinical application and interpretation (Siddaway et al., 2021). Our results could help in the development of intervention in late‐life suicide, not only as biological marker for late‐life suicidality, but also as potential target for brain stimulation. Machine learning has made its foray into suicide research; thus, more research must be conducted to consolidate our results with scalable implementation in clinical setting.

## AUTHOR CONTRIBUTIONS

*Conceptualization; data curation; formal analysis; funding acquisition; investigation; methodology; supervision; validation; visualization; writing—original draft; writing—review and editing*: Chemin Lin. *Conceptualization; data curation; formal analysis; investigation; resources; software; supervision; validation; visualization; writing—original draft; writing—review and editing*: Chih‐Mao Huang. *Formal analysis; methodology; software; validation; visualization; writing—original draft; writing—review and editing*: Wei Chang. *Data curation; formal analysis; software; visualization; writing—review and editing*: You‐Xun Chang. *Conceptualization; supervision; writing—review and editing*: Ho‐Ling Liu. *Data curation; project administration; writing—original draft; writing—review and editing*: Shu‐Hang Ng. *Conceptualization; investigation; project administration; writing—original draft; writing—review and editing*: Huang‐Li Lin. *Conceptualization; validation; writing—original draft; writing—review and editing*: Tatia Mei‐Chun Lee. *Conceptualization; funding acquisition; investigation; methodology; project administration; resources; writing—original draft; writing—review and editing*: Shwu‐Hua Lee. *Conceptualization; data curation; formal analysis; investigation; methodology; project administration; resources; software; supervision; validation; visualization; writing—original draft; writing—review and editing*: Shun‐Chi Wu.

## CONFLICT OF INTEREST STATEMENT

The authors declare no conflicts of interest.

### PEER REVIEW

The peer review history for this article is available at https://publons.com/publon/10.1002/brb3.3348.

## Supporting information

Supp information

## Data Availability

fMRI Data and code to calculate cross‐sample entropy are available upon request.
