# Supplementary material for: Predicting suicidality in late‐life depression by 3D convolutional neural network and cross‐sample entropy analysis of resting‐state fMRI
Source: Brain Behav. 2024 Jan 15;14(1):e3348. doi: 10.1002/brb3.3348 (PMC10790060; doi:10.1002/brb3.3348)

| Supplement table 1. Demographic data and between group comparisons | | | | | | |
| --- | --- | --- | --- | --- | --- | --- |
|  | NS | Suicidality | SI | SA | Statistics | post-hoc |
|  | (n=35) | (n=48) | (n=26) | (n=22) |  |  |
| Age | 67.0±5.9 | 65±4.8 | 66.4±5.1 | 63.4±4.0 | F=3.4 * | NS > SA |
| Sex, (M/F) | 7/28 | 8/40 | 6/20 | 2/20 | Chi=2.3 |  |
| Education | 8.0±2.9 | 9.2±3.6 | 9.0±3.7 | 9.3±3.5 | F=1.6 |  |
| Disease course | |  |  |  |  |  |
| Onset | 57.1±7.9 | 50.4±11.6 | 52.8±12.1 | 47.9±10.8 | F=5.6* | NS > SA |
| Episodes | 1.9±1.6 | 3.3±2.9 | 2.8±1.6 | 3.7±3.8 | F=3.7* | SA > NS |
| ATHF load | 3.4±1.2 | 3.8±1.1 | 3.8±1.1 | 3.8±1.1 | F=1.1 |  |
| Psychological scales | |  |  |  |  |  |
| HAMD | 7.9±5.1 | 9.4±6.4 | 10.9±7.2 | 7.8±5.0 | F=3.2 * |  |
| BSS | 2.4±2.7 | 6.3±5.6 | 4.4±5.1 | 8.3±5.6 | F=8.3 ** | SA>NS, SA > SI |
| MMSE | 27.7±1.5 | 27.9±1.3 | 27.9±1.3 | 28.3±1.1 | F=0.9 |  |
| EC, Elderly control; NS, Non-suicidal Late-life depression; SI, Suicide ideator; SA, Suicide attempter; ATHF, antidepressant treatment history form; HAMD, 17-item Hamilton Depression Scale; BSS, Beck Scale for Suicide Ideation; BIS, Barratt Impulsiveness Scale; SPS, SAD PERSONS scale; CTQ, Childhood Trauma Questionnaire; MMSE, Mini-mental Status Examination; DSST, Digit symbol substitution test; DF, Digit forward; DB, Digit backward; NLS, Number letter sequencing; VF, Verbal fluency; FM, Facial memory; * p < 0.05; ** *p* < 0.005; *** *p* < 0.001; Bonferroni correction in post-hoc analysis. | | | | | | |

Supplement table 2. The six cross-validation results. The 11 patients without suicidality were mis-classified as having suicidality shown in gray background in the table. Notice that they were mostly low in probability (poll). PPV, positive predictive value.


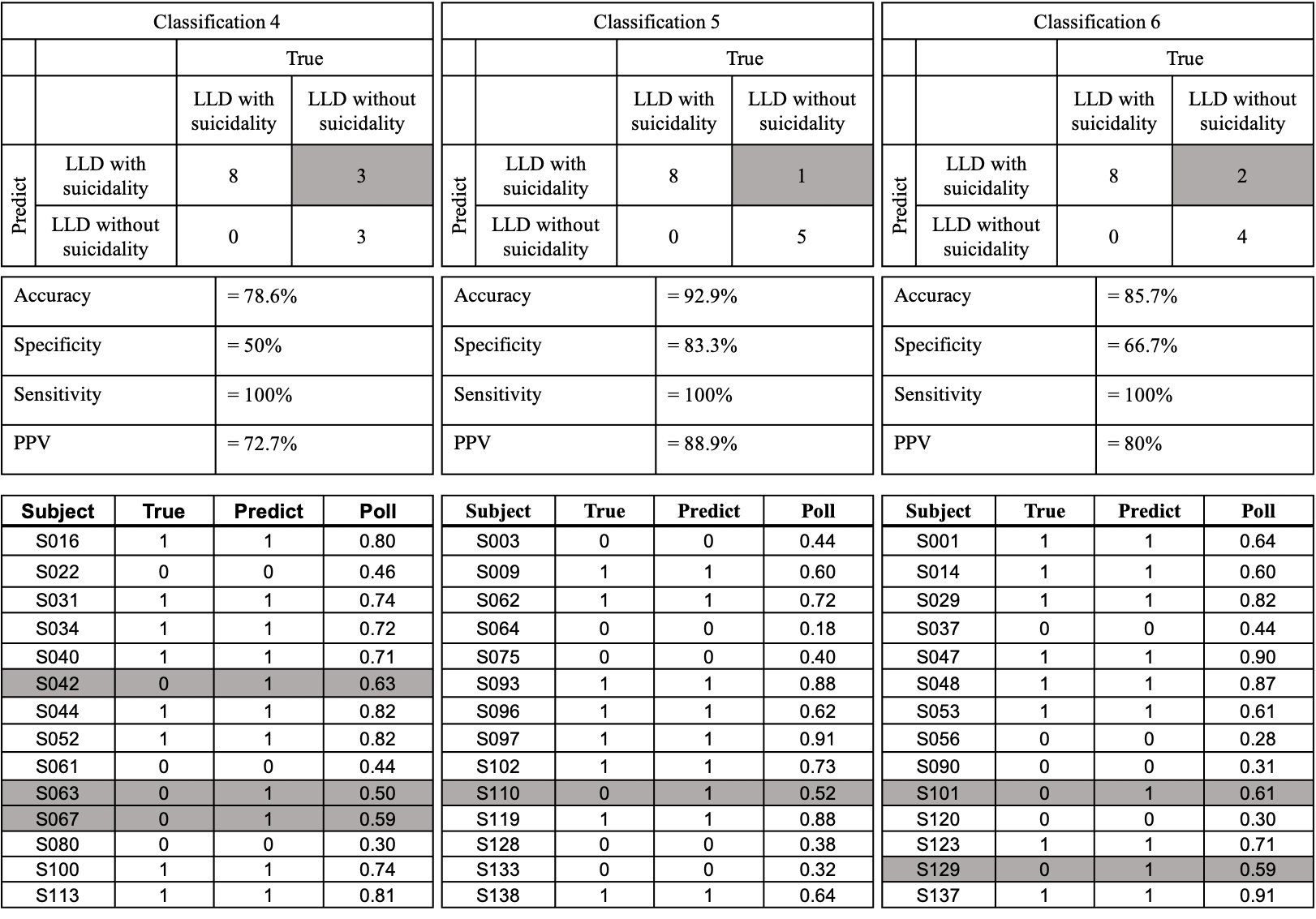

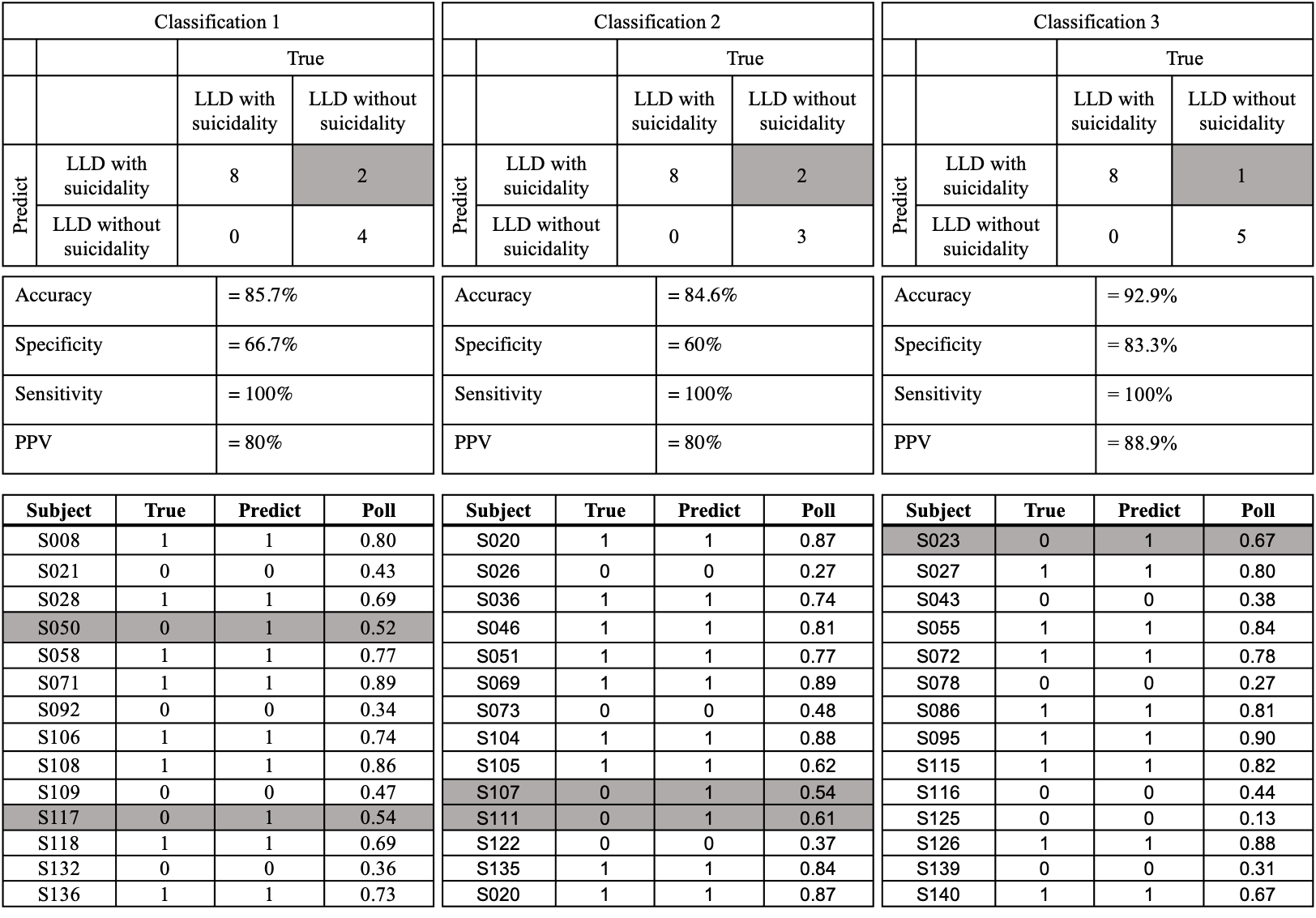

Supplement: Supplementary file 1 — Supp information [file BRB3-14-e3348-s001.docx]
